# Pulsation-limited oxygen diffusion in the tumour microenvironment

**DOI:** 10.1038/srep39762

**Published:** 2017-01-03

**Authors:** Edoardo Milotti, Sabrina Stella, Roberto Chignola

**Affiliations:** 1 Department of Physics, University of Trieste, Via Valerio 2, I-34127 Trieste, Italy; 2 Department of Biotechnology, University of Verona, Strada Le Grazie 15, CV1 I-37134, Verona, Italy

## Abstract

Hypoxia is central to tumour evolution, growth, invasion and metastasis. Mathematical models of hypoxia based on reaction-diffusion equations provide seemingly incomplete descriptions as they fail to predict the measured oxygen concentrations in the tumour microenvironment. In an attempt to explain the discrepancies, we consider both the inhomogeneous distribution of oxygen-consuming cells in solid tumours and the dynamics of blood flow in the tumour microcirculation. We find that the low-frequency oscillations play an important role in the establishment of tumour hypoxia. The oscillations interact with consumption to inhibit oxygen diffusion in the microenvironment. This suggests that alpha-blockers–a class of drugs used to treat hypertension and stress disorders, and known to lower or even abolish low-frequency oscillations of arterial blood flow –may act as adjuvant drugs in the radiotherapy of solid tumours by enhancing the oxygen effect.

It is now well recognized that solid tumours are not just ensembles of cancer cells but complex heterogeneous mixtures of malignant and non-malignant cells which are structurally and biochemically supported by an extracellular matrix of polysaccharides and fibrous proteins, and fed by an irregular network of blood vessels^1^. The tumour vascular network differs substantially from that of normal tissues^2^. For example, the architecture of tumour blood vessels is often more tortuous and irregular and the endothelial linings and basement membranes are incomplete or absent causing irregular blood flow and vascular leakiness^2^. The intervascular distance is large and this conspires with irregular blood flow to reduce oxygen and nutrient delivery to cells with the resultant development of hypoxic or even anoxic inner areas^2^,^3^,^4^,^5^. In their search for nutrients, tumour cells often wrap around blood vessels to form cords of living cells, and the consumption of oxygen, nutrients and eventually of drugs is not homogeneously distributed in solid cancers. Finally, lymphatic vessels are almost absent^6^; this means that, since the fluids are not drained and toxic molecules such as lactate are not removed from the environment, the chemical composition of interstitial fluids becomes abnormal, producing acidosis, and the pressure of interstitial fluids (IFP) increases. The increased IFP generates a net outward fluid flow which is believed to hinder the penetration of therapeutic drugs. Finally, acidosis can directly impair the action of several therapeutic drugs^1^,^6^,^7^. Hypoxia induces significant genomic and proteomic changes in tumour cells. A pivotal role in the hypoxic response of the cells is played by the Hypoxia Inducible Factor-1 (HIF-1). In well oxygenated cells, the cytoplasmic HIF-1α subunits bind the von Hippel-Landau protein, a component of an E3 ubiquitin ligase, that ultimately targets HIF-1α for proteasomal degradation. Under hypoxia, HIF-1α translocates to the nucleus and binds the HIF-1β subunit to form an active protein which is capable of turning on the expression of target genes upon binding to hypoxia responsive elements^2^,^3^. Among such genes are those coding for proteins that stimulate angiogenesis (e.g., VEGF, iNOS), cell proliferation and survival (e.g., EGF, IGF-2) and promote metabolic adaptation by increasing glucose uptake and utilization (e.g., through GLUT-1, Hexokinase). It has been shown that hypoxia can also induce genomic instability by increasing the mutation frequency of cells^2^,^3^. The highly selective tumour microenvironment can then promote the growth of more aggressive tumour phenotypes^2^,^3^,^8^. Thus, hypoxia is central to tumour evolution, growth, invasion and metastasis. In addition, hypoxia is known to reduce the efficacy of radiotherapy^9^,^10^. More generally, the structure of the tumour microenvironment has an adverse effect on many therapies. This structure hinders the diffusion of oxygen, nutrients and drugs, and the gradients that form in the microenvironment provide ecological niches that are filled by different cell variants^11^. The distribution of cells, in turn, alters the environment bringing about a complex feedback loop. Evident correlations exist between the aggressiveness of a tumour and the structure of its microenvironment, and the factors that characterize the microenvironment–such as IFP and hypoxia–can be used as prognostic indicators^2^,^3^,^4^,^10^. The existence of such correlations suggests that by modifying the microenvironment we can control the course of the disease: to this end much experimentation has been carried out to remodel the blood vessel network both with angiogenic and anti-angiogenic factors^12^.

Surprisingly, in spite of its importance for tumour biology and the clinical course of cancer disease, our understanding of the quantitative aspects of oxygen diffusion in the tumour microenvironment is not complete. Oxygen concentrations in tumour tissues are described by means of appropriate mathematical models that synthesize current knowledge on oxygen diffusion and consumption, see, e.g. ref. 13. Mathematical models of hypoxia have been used in attempts to predict the growth and the spread of tumour cells to surrounding tissues in patients starting from MRI scans of cancer lesions^14^. In Fig. 1 we compare the output of a stationary reaction-diffusion model with actual experimental data. The measurements of oxygen concentration deviate conspicuously from model predictions, and this means that we still miss some important detail of oxygen diffusion in solid tumours.

Hypoxia shows up differently in different tumours and even in different parts of individual tumours, where it is heterogeneous both in space and in time^15^,^16^. Stationary reaction-diffusion models do not take into account the spontaneous oscillations of the parameters of the cardiovascular system^17^,^18^, and indeed, besides the periodic oscillations due to breathing rhythms, fluctuations much slower than respiration of arterial pressure–such as the Mayer waves^18^–are known, and they are caused by the activity of the autonomous nervous system. These fluctuations propagate to the microcirculation, and in tumours the spontaneous oscillations of blood pressure and of blood volume are likely to affect oxygen concentration and delivery to the tumour microenvironment. Fluctuations of blood flow and oxygen concentration have been measured in mice using laser-Doppler flowmetry and recessed-tip oxygen microelectrodes in both normal tissues and solid tumours^19^. Fourier analysis has revealed that in tumours the magnitude of the fluctuations is higher than in normal tissue and that the power spectra display significant low-frequency oscillations in the range between 0 to 0.16 Hz (in mice heart and breathing frequencies occur at approximately 0.8–1 Hz and 5–6.6 Hz, respectively). These observations have been used to calculate probable distribution of oxygen enhancement ratios within a tumour, and then to compute the tumour control probability of radiation treatments^20^. It turns out that oxygen fluctuations may adversely affect hypofractionated radiation treatment schemes, like stereotactic radiosurgery and intraoperative radiotherapy^20^. Oxygen waves may have important implications for tumour hypoxia and therapy–as suggested by Braun *et al*.^19^–and to prove their role the standard reaction-diffusion models must be non-trivially extended to include both the oxygen fluctuations and the distribution of oxygen-consuming cells in the tumour tissue, and their outputs finally compared with actual data.

## Results

### Main results

**Figure 1:**
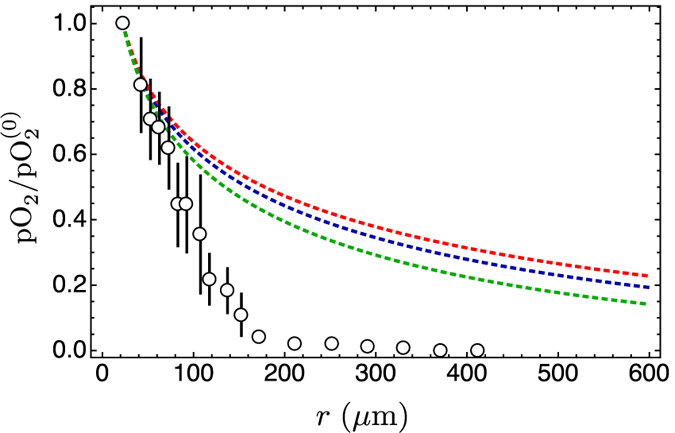
Comparison of model predictions with experimental data. The experimental data have been redrawn from Fig. 3 in ref. 35 and refer to measurements of partial oxygen pressure (pO_2_) in the tumour interstitium as a function of the distance from blood vessels (circles; bars represents the s.e.m. calculated from 15 samples). Here pO_2_ has been normalised with respect to the central value in the nearest blood vessel (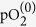). The lines represent the stationary solutions obtained from a reaction-diffusion model–discussed further on in the main text–with different values of the oxygen diffusion coefficient and with a measured consumption rate. Blue line: measured value of the diffusion coefficient in the tumour interstitium; red line: diffusion coefficient of oxygen in water, almost twice larger than the diffusion coefficient in the interstitium; green line: diffusion coefficient halved with respect to measured value in the interstitium; the oxygen consumption coefficient is taken as the measured value in refs 28, 29, 30. It is clear that the inadequacy of the stationary solutions does not depend on the variability of the diffusion coefficient.

Using a time-dependent oxygen supply in the context of a reaction-diffusion model, with the assumption of a marked spatio-temporal tumour heterogeneity, we find that the model successfully describes the fast decay of oxygen concentration in Fig. 1, and that this result is robust with respect to changes of the parameter values. In the following paragraphs we describe the mathematical development of the model. The details are given in the Methods section, and further considerations can be found in the Supplementary Information (SI).

### Mathematical model

**Figure 2:**
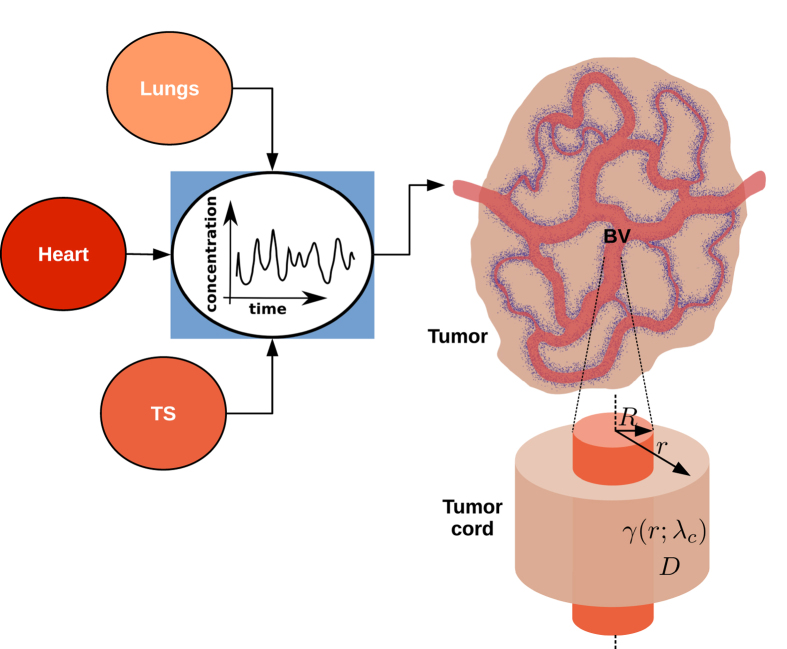
Schematic view of the factors affecting the perfusion of molecules in the tumour microenvironment. In mature cancers, tumour cells often wrap around blood vessels (BV) to form tumour cords. Oxygen, nutrient and drug availability is not constant in time, but is affected by breathing, by heart beat and by treatment schedules (TS), and by other physiological processes.

The dynamics of tumours spans many timescales, and here we consider times that range from about 1 s to one day. This means that the fast dynamics of molecular reactions contributes only with average values, while the growth of the tumour mass can be neglected and taken as fixed. Moreover, here we consider only small spatial regions, close to the capillary vessels, where diffusive phenomena are dominant. This setting is illustrated in Fig. 2, and the concentration Φ of any substance can be described by the generic reaction-diffusion equation


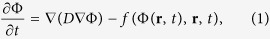


where the diffusion coefficient *D* can depend on position, and where the reaction term *f* can depend both on space and time, implicitly by way of a dependence on concentration, or explicitly. Here we assume that *D* is nearly independent of position, and that the reaction term *f*(Φ(**r**, *t*), **r**, *t*) ≈ *γ*Φ, as discussed in the Methods section, so that the reaction-diffusion equation (1) becomes a linear partial differential equation


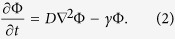


In this way we can utilize the superposition principle and the usual extension of the solutions to the complex domain as an aid in solving the equations. The superposition principle also helps in understanding complex, realistic situations, as shown in a very close context by the “Green’s function formalism” of Secomb and others^21^. Then we find solutions of equation (2) using the complex Fourier expansion of the concentration in the time domain


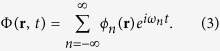


**Figure 3:**
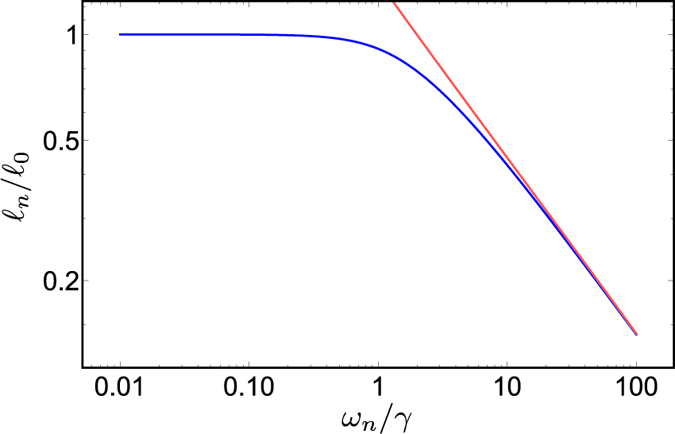
Log-log plot of 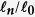 vs. *ω_n_*/*γ* (blue line). The curve is essentially flat for *ω*_*n*_ < *γ*, while it approaches the power law 1/*ω*^1/2^ (red line) for *ω*_*n*_ > *γ*. The figure shows that *γ* marks the transition between the two regimes, while the actual value of 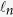 also depends on the *D*/*γ* ratio. In the absence of consumption the decay length would depend on frequency only, and it would follow the power law.

It is useful to consider the planar case first – even though it is not directly relevant to physiology – because it sets the scene for further developments. The main result is that every Fourier component decays exponentially as one moves away from the plane interface where oxygen is fed into a half-space, and the decay lengths are frequency dependent


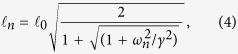


where 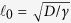. The decay lengths 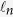 are largest for *ω*_*n*_ = *ω*_*0*_ = 0, and they decrease rapidly as *ω*_*n*_ grows larger than *γ*, as shown in Fig. 3. The figure also shows that *γ* marks the transition between the two regimes: when *ω*_*n*_ > *γ* the consumption rate becomes irrelevant, but when *ω*_*n*_ < *γ* the decay length is nearly independent of *ω*_*n*_ and much lower than the case without consumption.

### Cylindrical geometry

Even though a planar geometry has no meaning in a physiological context, it turns out that the considerations on decay lengths retain their importance also in a more realistic setting, i.e., when we consider the reaction-diffusion problem with a cylindrical geometry. In the vicinity of a blood vessel the oxygen concentration depends only on the distance *r* from its center if we neglect the dependence in the axial direction, which is due to the slow oxygen depletion of erythrocytes as they move along vessels. Then, the solutions of the reaction-diffusion equation close to a blood vessel of radius *R* are modified Bessel functions of the second kind with a complex argument^22^: 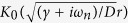. Asymptotically these solutions decay faster-than-exponentially, i.e, as the product of an exponential, with the same decay length as the planar case, times 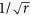. Again, in the stationary case (*ω*_*n*_ = *ω*_*0*_ = 0) the falloff is slowest, and the decay length is longest.

**Figure 4:**
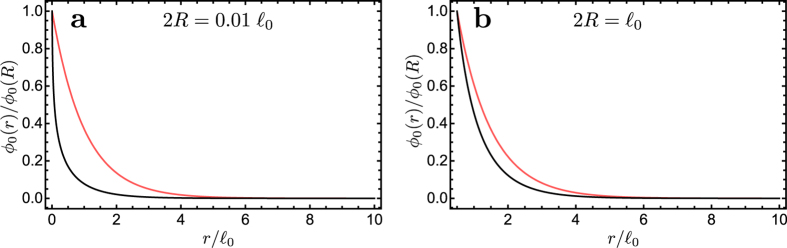
Plots of *ϕ*_0_(r) vs. 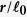 for different values of the blood vessel diameter 2R and with 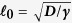 in the case of cylindrical geometry. The black line in each panel is the solution *ϕ*_0_(*r*), while the red line curve is the exponential 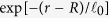. The solution *ϕ*_0_(*r*) is always subexponential, however it approaches the exponential as the diameter of the blood vessel increases.

A numerical evaluation of the solution shows that close to the blood vessel the falloff depends on the blood vessel radius *R*. The behaviour of the solution *ϕ*_0_(*r*) in the stationary case (*ω*_*n*_ = *ω*_*0*_ = 0) for different vessel sizes is illustrated in Fig. 4: if the diameter is smaller than the decay length 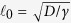, then the solution *ϕ*_0_(*r*) decays considerably faster than the corresponding exponential that we found in the solution for the half-space, and the effect is quite large for small diameter values. For example, with a diameter which is 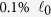, the solution *ϕ*_0_(*r*) decreases to half its value on the boundary surface at a distance which is 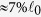, while the exponential falls to half its initial value at a distance 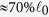.

Remarkably, the rapid decrease due to the faster-than-exponential decay of the stationary solution *ϕ*_0_(*r*) is not sufficient to account for the observed data, as shown by the curves in Fig. 1, which compares the behaviour of *ϕ*_0_(*r*) for different values of the diffusion coefficient with actual measurements. This result hints at the importance of the physiological oxygen fluctuations. However, before taking the fluctuations into account we extend our description of the microenvironment close to a blood vessel.

### Tumour cords

Tumours have a nonuniform structure, and in some cases tumour cells grow along capillary vessels and form tumour cords^5^,^23^ (see Fig. 2). To take this structure into account, we assume that the viable cell density decreases roughly exponentially with increasing distance from the nutrient supply system as discussed in refs 24 and 25 and that the local oxygen consumption is proportional to cell density. This means that the consumption rate *γ* is a decreasing function of the radius *r*


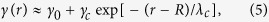


where *R* is the radius of the capillary vessel, and *r* > *R*. Since there is a decreasing number of tumour cells further away from the blood vessel, the decrease of oxygen concentration should be less sharp than in the case examined in the previous section.

Because of the nonuniform cell density, the differential equation becomes more complex, and it does not have a closed-form solution. However it is still possible to express the solution as an infinite product, and turning to logarithms it takes on a manageable form, still involving modified Bessel functions with complex argument


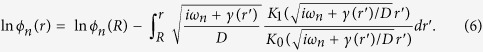


**Figure 5:**
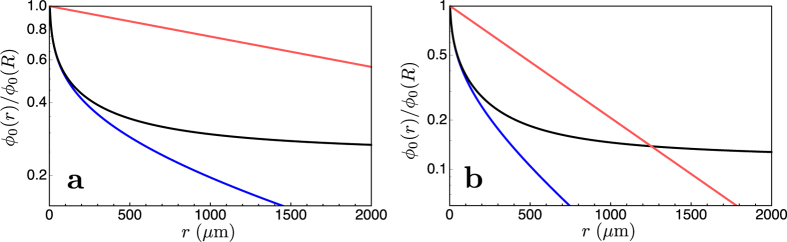
Semilog plots of the normalized oxygen concentration *ϕ*_0_(*r*)/*ϕ*_0_(*R*) vs. *r* (μm) in the case of tumour cords. The black line in each panel is the solution for tumour cords, eq. (6) with λ_c_ = 120 μm. The blue line shows the concentration with a fixed consumption rate *γ* = *γ*_*0*_ + *γ*_*c*_; the red line is the exponential 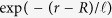 with 
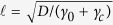
. All curves are calculated with blood vessel diameter 2*R* = 6 μm. The panels illustrate the combinations of parameters that maximize or minimize the characteristic length 
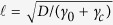
 taking the values given in the text. Left panel, case with the smallest consumption coefficient (*γ*_*0*_ = 0; *γ*_*c*_ = 1.66 10^−4^s^−1^; *D* = 2000 μm^2^/s); *ϕ*_0_(*r*)/*ϕ*_0_(*R*) = 0.5 at *r* ≈ 118 μm; at large radius *ϕ*_0_(*r*)/*ϕ*_0_(*R*) ≈ 0.25. Right panel, case with the largest consumption coefficient (*γ*_*0*_ = 0; *γ*_*c*_ = 5 10^−3^ s^−1^; *D* = 2000 μm^2^/s); *ϕ*(*r*)/*ϕ*_0_(*R*) = 0.5 at *r* ≈ 48 μm; at large radius *ϕ*_0_(*r*)/*ϕ*_0_(*R*) ≈ 0.12.

**Figure 6:**
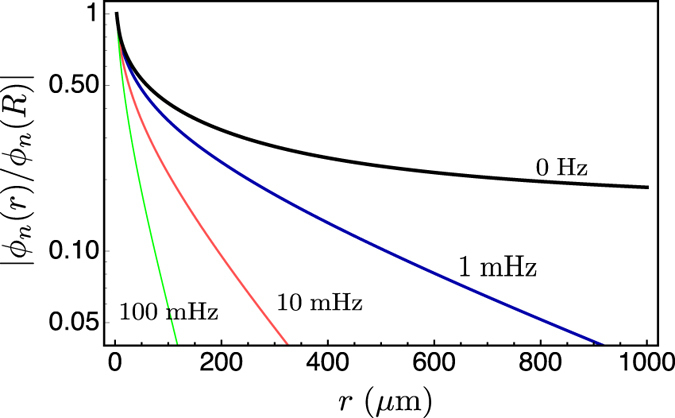
Plots of |*ϕ*_n_(*r*)/*ϕ*_n_(*R*)| vs. *r* (μm) in the case of a tumour cords, eq. (6), for different frequencies. All curves calculated with 2*R* = 6 μm., *D* = 2000 μm^2^/s, *γ*_*0*_ = 0, *γ*_*c*_ = 2.16 10^−3^ s^−1^, and *λ*_*c*_ = 120 μm. The black line is the stationary solution (*ω*_*0*_ = 0). Blu line, *ω*_*n*_ = 2π 0.001 s^−1^; red line, *ω*_*n*_ = 2π 0.01 s^−1^; green line, *ω*_*n*_ = 2π 0.1 s^−1^.

Figures 5 and 6 show the behaviour of the solution (6). All the parameters used in the numerical evaluation are extrapolated from experimental data and apply to solid tumours. We take the decay length of the exponential reduction of the consumption rate λ_c_ = 120 μm from refs 24 and 25, and the diffusion constant of oxygen as measured both in blood and tissues^26^,^27^) *D* = 2 × 10^−9^ m^2^/s. The rates of oxygen consumption in different areas of *in vivo* tumours have been elegantly and precisely measured, and they have been shown to vary in the range 1.66 10^−4^–5 10^−3^ s^−1^ (mean value 2.16 10^−3^ s^−1^)^28^,^29^,^30^. Finally, measurements on melanomas^31^ indicate that the average microvessel diameter is about 5 μm, i.e., *R* = 2.5 μm, just enough to let one erythrocyte through.

As expected, Fig. 5 shows that the solution *ϕ*_0_ has a fast initial decrease that closely follows that found in the cylindrical case, and deviates from it further away from the blood vessel, where the oxygen consumption rate becomes negligible because of the vanishing population of live tumour cells in the necrotic, hypoxic region.

Figure 6 displays the behaviour of the solution (6) in the time-dependent case. The fluctuations lead to a further reduction in range with respect to the stationary case. Here we have taken frequencies that have a pathophysiological meaning^19^, and we discuss them in the next section.

### Comparison with experimental data

**Figure 7:**
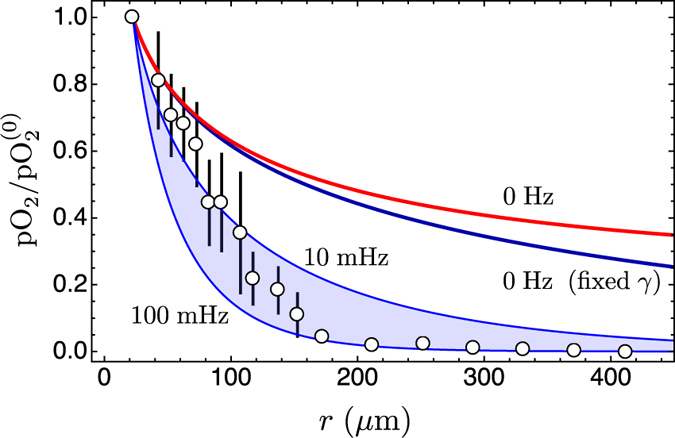
Comparison of model predictions with experimental data. The experimental data have been redrawn from Fig. 3 in ref. 35 and refer to measurements of partial oxygen pressure (pO_2_) in the tumour interstitium as a function of the distance from blood vessels (circles; bars represents the standard error calculated from 15 samples). Here pO_2_ has been normalised with respect to the central value in the nearest blood vessel (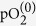); further details, including the choice of boundary conditions, are specified in the Supplementary Text. The blue line is the ratio *ϕ*_0_(*r*)/*ϕ*_0_(*R*) with a fixed consumption rate. The red line is the ratio *ϕ*_0_(*r*)/*ϕ*_0_(*R*), eq. (6), in the case of tumour cords, with a nonuniform consumption rate. The coloured band is delimited by the lines |*ϕ*_n_(*r*)/*ϕ*_n_(*R*)| that correspond to the limiting frequencies *ω*_*n*_ = 2π 0.1 s^−1^ (lower curve) and *ω*_*n*_ = 2π 0.01 s^−1^ (upper curve). These frequencies define the interval of the observed frequencies with the highest amplitude for oxygen oscillations in the tumour microcirculation^19^. The other parameters are listed in the main text. It should be noted that there are no free parameters and that the solutions of model equations are true predictions of the oxygen concentration in the tumour interstitium.

Experiments that probe the tumour microenvironment are extremely difficult, and there are only scant data in the current scientific literature. This is complicated by the large variability that exists between tumours, as far as tumour histology and pO_2_ profiles are concerned. The tumour tissue can be compact or wrapped around vessels as tumour cords with wide necrotic regions in between, and pO_2_ can be very low in the tumour center only or also in its periphery^15^,^16^,^32^, and can also display spatial heterogeneity in an individual tumour. Moreover, this spatial heterogeneity combines with temporal variability on time scales of several tens of minutes^16^,^19^. Finally, in tumours *in vivo* the oxygen consumption rate spans the range 1.66 10^−4^–5 10^−3^ s^−1^ (see refs 28-30, 29, 30) (this is very different in cultured tumour cells^33^ where the rate is contained in the interval 0.0078–1.9 s^−1^). Before attempting a comparison with actual measurements, we list a few distinct classes on the basis of the experimental observations in refs 15, 16, 19, 28, 32 and 34 and references cited therein, and we point out, at least qualitatively, the correspondence between such observations and our mathematical description of tumour hypoxia. We attempt to integrate the information from histological/biochemical analyses^15^,^28^,^32^ with known facts about oxygen flow in solid tumours^16^,^19^,^34^. In this way we hope to make clear how the models discussed above can help treating the extremely heterogeneous aspects of oxygen availability in solid tumours. We identify five classes:

1. *compact tumor tissue, low consumption rate, quasi-regular oxygen flow*: in this case diffusion is unhindered, oxygenation is at a nearly normal level, and even parts of the tumour tissue that are relatively far from blood vessel can get a sufficient supply of oxygen. This situation can be modelled by means of equations (13),(14),(15),(16),(17),(18).
2. *compact tumour tissue, medium/high consumption rate, little necrosis far from blood vessels, quasi-regular oxygen flow*: with a medium/high consumption rate, those cells that are close to blood vessels subtract oxygen from areas that lie further away from blood vessels, and lead to the formation of distant hypoxic regions. In this case the amplitude of the constant term in the Fourier expansion of the measured pO_2_ signal is large, and this situation can be modelled again by means of equations (13),(14),(15),(16),(17),(18) (see, e.g., the curve marked “0 Hz (fixed γ)” in Fig. 7, as discussed further below).
3. *tumour cords, high consumption rate, extended necrosis far from blood vessels, quasi-regular oxygen flow*: this is a case similar to the previous one, but the presence of cells wrapped around blood vessels and of extended areas composed of dead cells between tumour cords confines oxygen consumption within a limited distance (a few cell layers) from blood vessels. Thus, elevated oxygen concentrations can be measured in the inner tumour regions. Mathematically, this means that the amplitude of the constant term in the Fourier expansion of the measured pO_2_ signal is higher than that of case 2. This situation can be modelled by means of equations (19)–(20) (see, e.g., the curve marked “0 Hz” in Fig. 7).
4. *compact tissue/tumor cords, low consumption rate, little necrosis far from blood vessels, bursting oxygen flow (widely separated reoxygenation events)*: widely separated reoxygenation events correspond to a very low average oxygen tension, and the fluctuating part of the oxygen signal is strongly attenuated even by a low consumption rate. This causes very low oxygen levels in distal regions. Mathematically, in this case the amplitude of the constant term in the Fourier expansion of the pO_2_ signal is very low when compared with the amplitude of the lowest harmonics, and the signal amplitude is reasonably well represented by the absolute value of the amplitude of the first harmonic. This situation can be modelled once again by means of equations (13),(14),(15),(16),(17),(18).
5. *compact tissue/tumor cords, low consumption rate, extended necrosis far from blood vessels, bursting oxygen flow (widely separated reoxygenation events)*: general behaviour as in the previous case, however here the constant term may be higher because of the reduced oxygen consumption due to necrosis. This situation can be modelled by means of equations (19)–(20) (see, e.g., the coloured band in Fig. 7).

Unfortunately in the current literature there are no experiments that give both an accurate evaluation of the partial oxygen pressure at a given distance from a blood vessel and its dynamics, therefore for a comparison we use averaged data without an explicit time dependence^35^. In the experiment of Helmlinger, Yuan, Dellian and Jain^35^ the partial pressure of oxygen in the tumour interstitium was measured at high spatial resolution (≤10 μm) *in situ* in a human tumour xenograft using phosphorescence quenching microscopy. They report an oxygen tension far from selected blood vessels that is extremely low, and therefore these data cannot belong to case 1, and can possibly be explained only in a stationary model with a very high oxygen consumption rate, or in the context of a bursting oxygen flow with a moderate consumption rate. Figure 7 provides model predictions that correspond to case 5, with the parameters of the previous section and with the range of frequencies *ω*_*n*_ = 2π 0.1 s^−1^ and *ω*_*n*_ = 2π 0.01 s^−1^ of the pathophysiological rhythms given in ref. 19, and with blood vessel radius *R* = 22.5 μm (Helmlinger *et al*.^35^ report blood vessel diameters in the range 10–80 μm and 22.5 μm is the median radius). The figure shows that the measured pO_2_ in ref. 35 compares very well with the predictions of the model with fluctuating oxygen tension and tumour cords.

**Figure 8:**
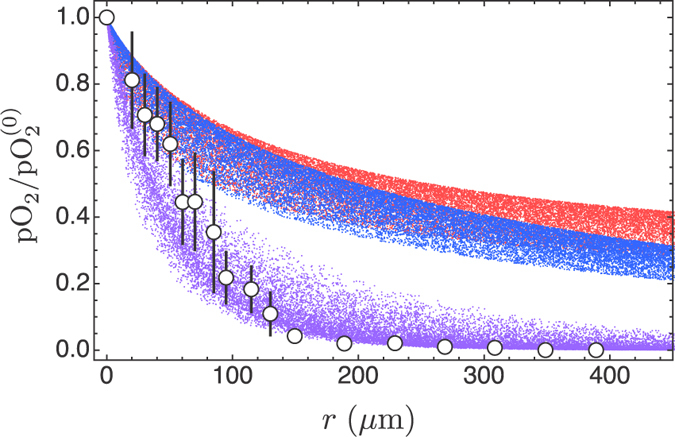
Monte Carlo simulation of model predictions that takes into account both the spread of frequencies and the spread of blood vessel radii (in the stationary cases, only the random distribution of the blood vessel radius is taken into account). Again, we plot pO_2_ normalised with respect to the central value in the nearest blood vessel (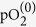); the calculation has been repeated 20000 times for the stationary case (blue dots), the stationary case with tumour cords (red dots), and the case of bursting oxygen flow (violet dots).

The dependence on blood vessel radius is nonlinear, and the choice of the median value *R* = 22.5 μm is open to question. To settle this problem we carried out a Monte Carlo simulation where we draw the blood vessel radius from a uniform distribution with the range 5–40 μm, and the frequency *ω*_*n*_ from a uniform distribution with the range 2π 0.1 s^−1^–2π 0.01 s^−1^. We use uniform distributions because they are the least informative and we are in a situation where nothing is known about the actual distributions of frequencies and radii. We also draw the distance from the blood vessel from a uniform distribution with the range 0–400 μm to span the same range shown in Fig. 7. The results of the simulation are shown in Fig. 8.

**Figure 9:**
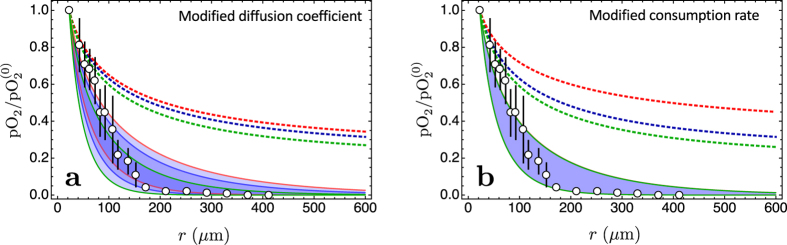
Robustness of the results shown in Fig. 7. Left panel: same parameters as Fig. 7 except for the diffusion coefficient. The original belt is bounded by the blue lines, the red lines delimit a belt that corresponds to a larger value of the diffusion coefficient (*D* = 3200 μm^2^/s, as in pure water), the green lines refer to a smaller diffusion coefficient (*D* = 1000 μm^2^/s). Right panel: same parameters as Fig. 7 except for the consumption rate. Here the consumption rate covers the range of values measured *in vivo* by Diepart *et al*.^28^,^29^,^30^. The original belt of Fig. 7 is nearly unchanged. In both figures the dotted lines represent the stationary solution (0 Hz) for tumour cords.

**Figure 10:**
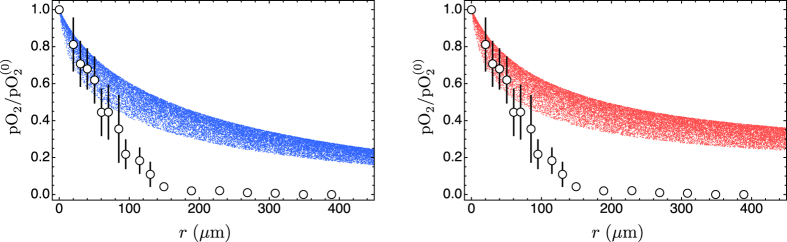
Monte Carlo simulation of model predictions that takes into account the spread of blood vessel radii for the stationary solutions for compact tissue (left panel, blue) and tumour cords (right panel, red). Here we take the highest oxygen consumption rate measured in refs 28, 29, 30, *γ* = 0.005 s^−1^.

These results are quite robust with respect to changes of the model parameters, as demonstrated by the curves in Fig. 9. The coloured bands in the figure represent the frequency region 10 mHz–100 mHz, and we see that they are remarkably stable under changes of the diffusion coefficient and of the consumption rate. On the contrary, the stationary solutions are much less stable. Finally, the comparison with the stationary solutions does not change when we consider the spread of blood vessel radius, as shown in Fig. 10.

## Discussion

In the introduction we noted that there are many factors that appear to affect the observed hypoxia and contribute to the effective barrier to the penetration of many chemicals into the tumour microenvironment. Our results indicate that the distribution of oxygen close to the smallest blood vessels is determined by a combination of three important elements: the distribution of live cells around blood vessels, the consumption rate of oxygen in this microenvironment, and the pathophysiological rhythms that regulate the oxygen inflow. These rhythms are the most mysterious component in the trio. Low-frequency fluctuations of this kind stand out in physiology, and apart from the obvious variability in oxygen concentration due to breathing and to the heart rhythm, there are also low frequency oscillations (around 0.1 Hz) known as “Mayer waves” that are relevant, e.g., in the activity of the brain^18^. Oxygen oscillations at even lower frequencies (around 0.001– 0.01 Hz)–unrelated to the physiological rhythms–have also been measured in tumour tissues^16^,^19^,^36^,^37^. Our results demonstrate that the low frequency oscillations of oxygen concentration are quite effective in further limiting the penetration of oxygen deep into the tumour interstitium. Other factors like the structure of the extracellular matrix are not absent, they contribute to the specific values of the parameters that enter the reaction-diffusion equation–for instance, both the composition and the structure of the extracellular matrix determine the effective value of the diffusion coefficient *D*, while the metabolic activity and the extracellular environment determine the actual value of the consumption rate – but in this way they are relegated to a sort of minor role. The equations are quite robust with respect to changes of these parameters, as demonstrated by the curves in Fig. 9. The coloured bands in the figure represent the frequency region 10 mHz–100 mHz, and we see that they are remarkably stable under changes of the diffusion coefficient and of the consumption rate. On the contrary, the stationary solutions are much less stable.

It is known that in addition to the fluctuations of oxygen concentration there are also blood pressure fluctuations^38^: these barometric fluctuations may play a role in the reduced penetration of drugs and chemicals into solid tumours, and we plan to use this fact in an extension of this work. However, these are not the only direct clinical implications. We have shown that low-frequency oscillations strongly reduce the diffusion of oxygen in the tumour microenvironment, and tumour hypoxia is known to affect radiotherapy^9^,^10^. It has been found that the low-frequency rhythms of arterial circulation can be strongly attenuated, or even abolished, after acute alpha-adrenoreceptor blockade^18^,^39^. Alpha-blockers are well-known and well-tolerated drugs and they are already used to treat hypertension, anxiety and panic disorders, such as the post-traumatic stress disorder^40^,^41^,^42^. Our results suggest that their action on blood circulation may transiently improve the oxygenation of the tumour microenviroment, and therefore that they could be used as an adjuvant therapy in radiation treatment by enhancing the oxygen effect^10^,^43^.

## Methods

### Reaction-diffusion equations in the time-dependent regime

In the tumour microenvironment (see Fig. 2) active transport is mostly ruled out, and the concentration Φ of any substance can be described by the generic reaction-diffusion equation (1). In most cases the reaction term f(Φ(**r**, *t*), **r**, *t*) corresponds to a combined sum of enzyme-mediated reactions that are described by Michaelis-Menten quasi-stationary processes^44^


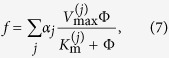


where all the coefficients can depend on the concentrations of other substances.

Throughout this paper we make the additional, simplifying assumption that 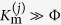, so that


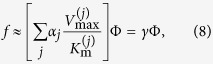


and the reaction-diffusion equation (1) becomes the linear partial differential equation (2). We find solutions of equation (2) using the complex Fourier expansion of the concentration in the time domain


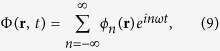


where *ω* is the fundamental frequency of the periodic fluctuations and where 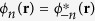 since the concentration is a real function.

It is useful to consider the planar case first–even though it is not directly relevant to physiology–where *ϕ*_n_ depends on a single space variable *x* and we take the boundary condition 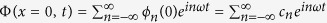. Then, we find that the Fourier coefficients satisfy the equations


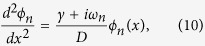


with *ω*_*n*_ = *nω* and for *x* > 0 (the details of the calculation are given in the Supporting Information (SI)). Using the boundary condition and the requirement that the solution never diverges in the subspace *x* > 0 we obtain a solution that has an oscillating part, and decays exponentially


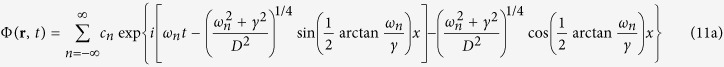



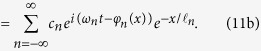


Equation (11a) shows that the decay lengths are


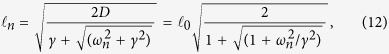


where 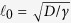 (see the SI for further details).

### Cylindrical geometry

In cylindrical coordinates (*r, θ, z*), the linearized reaction-diffusion equation (2) becomes


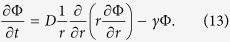


We separate again the spatial and the time variables, taking candidate solutions


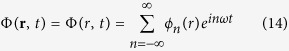


that lead to the equations for the Fourier coefficients


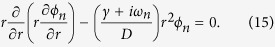


The boundary conditions are defined by the inner surface of the blood vessel (*r* = *R*, where *R* is the radius of the blood vessel), where we set 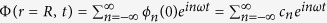, and by 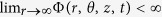.

The equations (15) are modified Bessel equations, and the solutions that satisfy the boundary conditions are the modified Bessel functions of the second kind *K*_*0*_(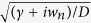
r). The complete solution for *r* ≥ *R* is


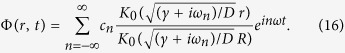


We can get a first glimpse into the behaviour of this solution noting that the asymptotic expansion of the modified Bessel function *K*_*0*_(*z*) is


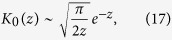


(see, e.g. ref. 45), where


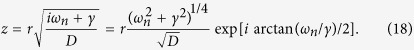


The asymptotic expansion shows that the exponential decay length is the same as that of the planar case, eq. (12), however here there is a faster-than-exponential falloff because of the 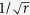 dependence. Again, in the stationary case (n = 0) the falloff is slowest, and the decay length is longest, 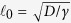.

### Tumour cords

Using the position-dependent consumption rate (5) the equations for the Fourier coefficients (15) become


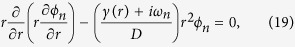


with the same boundary conditions as in the previous section.

Now the solution can be expressed as an infinite product (see the SI), the multiplicative structure becomes additive when we take logarithms, and the infinite product turns into an integral. The complete solution of equation (19) obtained with the method described in the SI is


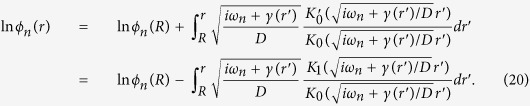


which is eq. (6) in the Results section.

## Additional Information

**How to cite this article**: Milotti, E. *et al*. Pulsation-limited oxygen diffusion in the tumour microenvironment. *Sci. Rep.* **7**, 39762; doi: 10.1038/srep39762 (2017).

**Publisher's note:** Springer Nature remains neutral with regard to jurisdictional claims in published maps and institutional affiliations.

## Supplementary Material

Supplementary Information
