# Supplementary material for: Pulsation-limited oxygen diffusion in the tumour microenvironment
Source: Sci Rep. 2017 Jan 3;7:39762. doi: 10.1038/srep39762 (PMC5206636; doi:10.1038/srep39762)
Supplement: Supplementary Information [file srep39762-s1.pdf]

Supporting Information for "Pulsation-limited oxygen diffusion in the tumour microenvironment."

Edoardo Milotti* and Sabrina Stella
Department of Physics, University of Trieste, Via Valerio 2, I-34127 Trieste, Italy

Roberto Chignola
Department of Biotechnology, University of Verona, Strada Le Grazie 15, CV1 I-37134, Verona, Italy

I. REACTION-DIFFUSION IN A HALF-SPACE

The diffusion equation with a simple linear reaction term writes:

$$\frac{\partial \Phi}{\partial t} = D\nabla^2\Phi - \gamma\Phi. \tag{S1}$$

In a planar geometry, ϕ_n depends on a single space variable x and we take the boundary condition $\Phi(x=0,t) = \sum_{n=-\infty}^{\infty} \phi_n(0)e^{in\omega t} = \sum_{n=-\infty}^{\infty} c_n e^{in\omega t}$. In this case, equation (S1) takes on the simpler form

$$\frac{\partial \Phi}{\partial t} = D\frac{\partial^2\Phi}{\partial x^2} - \gamma\Phi. \tag{S2}$$

We find solutions of equation (S2) using the complex Fourier expansion of the concentration in the time domain

$$\Phi(x,t) = \sum_{n=-\infty}^{\infty} \phi_n(x)e^{in\omega t}, \tag{S3}$$

where the usual relation $\phi_n(x) = \phi^*_{-n}(x)$ between pairs of Fourier coefficients holds, since the concentration is a real function. By substitution we find that the space-dependent Fourier coefficients satisfy the equation

$$\frac{d^2\phi_n}{dx^2} = \frac{\gamma + i\omega_n}{D}\phi_n(x) \tag{S4}$$

in the half-space $x > 0$, with $\omega_n = n\omega$. Therefore, when we let $\phi_n(x) = c_n e^{ik_n x}$ – so that $\Phi(x,t) = c_n e^{i\omega_n t + ik_n x}$ – we find

$$k^2 = -\frac{\gamma + i\omega_n}{D} = \frac{1}{D}\sqrt{\omega_n^2 + \gamma^2}\; e^{i\arctan \omega_n/\gamma + i\pi}, \tag{S5}$$

and

$$k = \pm\left(\frac{\omega_n^2+\gamma^2}{D^2}\right)^{1/4}\left[\sin\left(\frac{1}{2}\arctan\frac{\omega_n}{\gamma}\right) + i\cos\left(\frac{1}{2}\arctan\frac{\omega_n}{\gamma}\right)\right]. \tag{S6}$$

Finally, using the boundary conditions we obtain the solution

$$\Phi(x,t) = c_n \exp\left\{i\left[\omega_n t + \left(\frac{\omega_n^2+\gamma^2}{D^2}\right)^{1/4}\sin\left(\frac{1}{2}\arctan\frac{\omega_n}{\gamma}\right)x\right] - \left(\frac{\omega_n^2+\gamma^2}{D^2}\right)^{1/4}\cos\left(\frac{1}{2}\arctan\frac{\omega_n}{\gamma}\right)x\right\}, \tag{S7}$$

and from the trigonometric identity

$$\cos\frac{\arctan y}{2} = \sqrt{\frac{\cos(\arctan y)+1}{2}} = \sqrt{\frac{1+\sqrt{1+y^2}}{2\sqrt{1+y^2}}}, \tag{S8}$$

we find the decay length ℓ_n

$$\ell_n = \sqrt{\frac{2D}{\gamma + \sqrt{(\omega_n^2+\gamma^2)}}} = \ell_0\sqrt{\frac{2}{1+\sqrt{(1+\omega_n^2/\gamma^2)}}}. \tag{S9}$$

---

* milotti@units.it

II. CYLINDRICAL SYMMETRY

Assuming cylindrical symmetry, the reaction-diffusion equation in cylindrical coordinates is

$$\frac{\partial \Phi}{\partial t} = D \frac{1}{r} \frac{\partial}{\partial r}\left(r \frac{\partial \Phi}{\partial r}\right) - \gamma \Phi. \tag{S10}$$

We separate again the spatial and the time variables, taking candidate solutions

$$\Phi(\mathbf{r}, t) = \Phi(r, t) = \sum_{n=-\infty}^{\infty} \phi_n(r) e^{in\omega t}, \tag{S11}$$

that lead to the equations for the Fourier coefficients

$$r \frac{\partial}{\partial r}\left(r \frac{\partial \phi_n}{\partial r}\right) - \left(\frac{\gamma + i\omega_n}{D}\right) r^2 \phi_n = 0. \tag{S12}$$

The boundary conditions are defined by the inner surface of the blood vessel ($r = R$, where R is the radius of the blood vessel), where we set $\Phi(r = R, t) = \sum_{n=-\infty}^{\infty} \phi_n(0) e^{in\omega t} = \sum_{n=-\infty}^{\infty} c_n e^{in\omega t}$, and by $\lim_{r\to\infty} \Phi(r, \theta, z, t) < \infty$. Equations (S12) are modified Bessel equations, and the solutions that satisfy the boundary conditions are the modified Bessel functions of the second kind $K_0\left(\sqrt{(\gamma + i\omega_n)/D}\, r\right)$. The complex argument of the Bessel function can be written as follows

$$s = r\sqrt{\frac{i\omega_n + \gamma}{D}} = r \frac{(\omega_n^2 + \gamma^2)^{1/4}}{\sqrt{D}} \exp\left[\frac{i}{2} \arctan\left(\frac{\omega_n}{\gamma}\right)\right], \tag{S13}$$

and since both ω_n and γ are nonnegative, so that $0 \leq \arctan(\omega_n/\gamma) \leq \pi/2$ and $|\arg(s)| \leq \pi/4$, we can use the following asymptotic expansion for $K_0(z)$ (see, e.g., eq. (9.7.2) in [1])

$$K_0(s) \sim \sqrt{\frac{\pi}{2z}} e^{-s}, \tag{S14}$$

and we find that the asymptotic behavior of the solution is

$$|\Phi(r, \theta, z, t)| \sim \sqrt{\frac{\pi\sqrt{D}}{2r(\omega_n^2 + \gamma^2)^{1/4}}} \cos\left[\omega_n t + \left(\frac{\omega_n^2 + \gamma^2}{D^2}\right)^{1/4} \sin\left(\frac{1}{2}\arctan\frac{\omega_0}{\gamma}\right) r\right] \times \exp\left[-\left(\frac{\omega_n^2 + \gamma^2}{D^2}\right)^{1/4} \cos\left(\frac{1}{2}\arctan\frac{\omega_n}{\gamma}\right) r\right]. \tag{S15}$$

The oscillatory and the exponential parts are just like those of equation (S7), however the asymptotic expansion (S15) includes a factor $r^{-1/2}$ and it has a faster-than-exponential decay. Again, as in the case of equation (S7), the longest characteristic length corresponds to the stationary case ($\omega_0 = 0$), and taking the boundary condition $\Phi(r = R, \theta, z, t) = \Phi_0$ the solution is

$$\Phi(r, \theta, z, t) = \varphi(r) = \Phi_0 \frac{K_0\left(\sqrt{\frac{\gamma}{D}}\, r\right)}{K_0\left(\sqrt{\frac{\gamma}{D}}\, R\right)}. \tag{S16}$$

III. REACTION-DIFFUSION AROUND TUMOUR BLOOD VESSELS: THE CASE OF TUMOUR CORDS

Here we develop a method to solve the reaction-diffusion equation in a complex setting where the oxygen absorption rate is position-dependent, and we start with the simpler case of reaction-diffusion in a half space and without explicit time dependence.

A. Stationary case

1. Reaction-diffusion in a half-space

In the time-independent case the reaction-diffusion equation in a half-space with variable consumption rate takes on the form

$$D\frac{\partial^2\Phi}{\partial x^2} - \gamma(x)\Phi(x) = 0, \tag{S17}$$

with $x \geq 0$, with the boundary condition $\Phi(0) = \Phi_0$, and with

$$\gamma(x) \approx \gamma_0 + \gamma_c \exp(-x/\lambda_c). \tag{S18}$$

In general equation (S17) does not have a closed-form solution, however we can still find a solution as follows. We divide the x range in thin layers with thickness Δx so that we can use the fixed-γ, time-independent solution of equation (S2) in each layer, and we obtain

$$\Phi(x+\Delta x) \approx \exp\left[-\sqrt{\frac{\gamma(x)}{D}}\Delta x\right]\Phi(x), \tag{S19}$$

and therefore by iteration we get

$$\begin{aligned}
&\Phi(x+n\Delta x) \approx \\
&\approx \exp\left[-\sqrt{\frac{\gamma(x+(n-1)\Delta x)}{D}}\Delta x\right]\ldots\exp\left[-\sqrt{\frac{\gamma(x+\Delta x)}{D}}\Delta x\right]\exp\left[-\sqrt{\frac{\gamma(x)}{D}}\Delta x\right] \\
&\approx \exp\left[-\sum_{k=0}^{n-1}\sqrt{\frac{\gamma(x+k\Delta x)}{D}}\Delta x\right]\Phi(x).
\end{aligned} \tag{S20}$$

Then, taking the limit $\Delta x \to 0$, $n\Delta x \to z$, we find

$$\Phi(x+y) = \exp\left(-\int_0^y \sqrt{\frac{\gamma(x+z)}{D}}dz\right)\Phi(x), \tag{S21}$$

or also

$$\Phi(x) = \exp\left(-\int_0^x \sqrt{\frac{\gamma(z)}{D}}dz\right)\Phi_0. \tag{S22}$$

As a curiosity we note that with the assumed behavior $\gamma(x) = \gamma_0 + \gamma_c \exp(-x/\lambda_c)$ – an exponentially decreasing γ with largest value $\gamma_0 + \gamma_c$ on the interface plane at $x = 0$ – it is possibile to integrate the exponent in (S22), since a primitive function exist

$$\int \sqrt{a+be^{cx}}\, dx = \frac{2}{c}\left[\sqrt{a+be^{cx}} - \sqrt{a}\,\mathrm{arctanh}\left(\frac{\sqrt{a+be^{cx}}}{\sqrt{a}}\right)\right], \tag{S23}$$

so that finally we find the exact solution

$$\Phi(x) = \exp\left[-S(x)\right]\Phi_0, \tag{S24}$$

with

$$\begin{aligned}
S(x) &= \int_0^x \sqrt{\frac{1}{D}\left[\gamma + \gamma_c \exp(-z/\lambda_c)\right]}\, dz \\
&= -2\lambda_c\left(\sqrt{\frac{\gamma+\gamma_c\exp(-x/\lambda_c)}{D}} - \sqrt{\frac{\gamma}{D}}\,\mathrm{arctanh}\sqrt{1+\frac{\gamma_c}{\gamma}\exp(-x/\lambda_c)}\right) \\
&\quad + 2\lambda_c\left(\sqrt{\frac{\gamma+\gamma_c}{D}} - \sqrt{\frac{\gamma}{D}}\,\mathrm{arctanh}\sqrt{1+\frac{\gamma_c}{\gamma}}\right).
\end{aligned} \tag{S25}$$

2. Cylindrical symmetry

We find that the previous derivation can be adapted to the time-independent reaction-diffusion equation in cylindrical coordinates

$$\frac{1}{r}\frac{\partial}{\partial r}\left(r\frac{\partial\Phi}{\partial r}\right) - \gamma(r)\Phi(r) = 0, \tag{S26}$$

with

$$\gamma(r) \approx \gamma_0 + \gamma_c \exp[-(r-R)/\lambda_c]. \tag{S27}$$

We proceed as above, bearing in mind that in each thin cylindrical shell the local solution is proportional to $K_0(\sqrt{\gamma(r)/D}\, r)$, with $\gamma(r)$ nearly constant, and we find

$$\Phi(r+\Delta r) \approx \Phi(r)\frac{K_0[\sqrt{\gamma(r)/D}\,(r+\Delta r)]}{K_0[\sqrt{\gamma(r)/D}\,r]}. \tag{S28}$$

Iterating this local step we find

$$\Phi(r+2\Delta r) \approx \Phi(r)\frac{K_0[\sqrt{\gamma(r+\Delta r)/D}\,(r+2\Delta r)]}{K_0[\sqrt{\gamma(r+\Delta r)/D}\,(r+\Delta r)]}\;\frac{K_0[\sqrt{\gamma(r)/D}\,(r+\Delta r)]}{K_0[\sqrt{\gamma(r)/D}\,r]}, \tag{S29}$$

and finally

$$\Phi(r+n\Delta r) \approx \Phi(r)\frac{\prod_{k=0}^{n-1} K_0[\sqrt{\gamma(r+k\Delta r)/D}\,(r+(k+1)\Delta r)]}{\prod_{k=0}^{n-1} K_0[\sqrt{\gamma(r+k\Delta r)/D}\,(r+k\Delta r)]}. \tag{S30}$$

Eq. (S30) can also be written in the form

$$\Phi(r+n\Delta r) \approx \Phi(r)\prod_{k=0}^{n-1}\left\{1+\sqrt{\frac{\gamma(r+k\Delta r)}{D}}\frac{K_0'[\sqrt{\gamma(r+k\Delta r)/D}\,(r+k\Delta r)]}{K_0[\sqrt{\gamma(r+k\Delta r)/D}\,(r+k\Delta r)]}\Delta r\right\}, \tag{S31}$$

after expansion of the numerator for small Δr. It is also possible to proceed in an equivalent way taking the logarithm of equation (S30) first, so that

$$\begin{aligned}\ln\Phi(r+n\Delta r) \approx \ln\Phi(r) &+ \sum_{k=0}^{n-1}\ln K_0[\sqrt{\gamma(r+k\Delta r)/D}\,(r+(k+1)\Delta r)] \\ &- \sum_{k=0}^{n-1}\ln K_0[\sqrt{\gamma(r+k\Delta r)/D}\,(r+k\Delta r)] \qquad \text{(S32)} \\ \approx \ln\Phi(r) &+ \sum_{k=0}^{n-1}\sqrt{\frac{\gamma(r+k\Delta r)}{D}}\frac{K_0'\left[\sqrt{\gamma(r+k\Delta r)/D}\,(r+k\Delta r)\right]}{K_0\left[\sqrt{\gamma(r+k\Delta r)/D}\,(r+k\Delta r)\right]}\Delta r, \qquad \text{(S33)}\end{aligned}$$

and therefore

$$\ln\Phi(r+r') = \ln\Phi(r) + \int_0^{r'}\sqrt{\frac{\gamma(r+r'')}{D}}\frac{K_0'\left[\sqrt{\gamma(r+r'')/D}\,(r+r'')\right]}{K_0\left[\sqrt{\gamma(r+r'')/D}\,(r+r'')\right]}dr'', \tag{S34}$$

or equivalently

$$\ln\Phi(r) = \ln\Phi(R) + \int_R^r\sqrt{\frac{\gamma(r')}{D}}\frac{K_0'\left[\sqrt{\gamma(r')/D}\,r'\right]}{K_0\left[\sqrt{\gamma(r')/D}\,r'\right]}dr' = \ln\Phi(R) + \int_R^r d\ln K_0\left[\sqrt{\gamma(r')/D}\,r'\right]. \tag{S35}$$

Notice that if $\gamma(r) = \gamma$, we find

$$\ln \Phi(r) = \ln \Phi(R) + \int_R^r d \ln K_0 \left[\sqrt{\gamma/D}\, r'\right] = \ln \varphi(R) + \ln \frac{K_0\left[\sqrt{\gamma/D}\, r\right]}{K_0\left[\sqrt{\gamma/D}\, R\right]}, \tag{S36}$$

i.e., we recover

$$\Phi(r) = \Phi(R) \frac{K_0\left[\sqrt{\gamma/D}\, r\right]}{K_0\left[\sqrt{\gamma/D}\, R\right]}, \tag{S37}$$

which is the original solution with constant γ.

Using the integral representation (see, e.g., eq. (9.6.24) in [1])

$$K_\nu(z) = \int_0^\infty e^{-z\cosh t} \cosh(\nu t) dt, \tag{S38}$$

it is easy to see that $K_0'(z) = -K_1(z)$ (eq. (9.6.27) in [1]), and therefore we can also rewrite the integral (S35) as follows

$$\ln \Phi(r) = \ln \Phi(R) - \int_R^r \sqrt{\frac{\gamma(r')}{D}} \frac{K_1\left[\sqrt{\gamma(r')/D}\, r'\right]}{K_0\left[\sqrt{\gamma(r')/D}\, r'\right]} dr'. \tag{S39}$$

B. Time-dependent case with periodic oxygen fluctuations

When we consider the time-dependent case eq. (S26) is replaced by the equation

$$\frac{1}{r}\frac{\partial}{\partial r}\left(r\frac{\partial \phi_n}{\partial r}\right) - \left(\frac{\gamma(r) + i\omega_n}{D}\right)\phi_n = 0, \tag{S40}$$

with the boundary conditions $\Phi(r = R, t) = \sum_{n=-\infty}^{\infty} \phi_n(0) e^{in\omega t} = \sum_{n=-\infty}^{\infty} c_n e^{in\omega t}$, and $\lim_{r\to\infty} \Phi(r, \theta, z, t) < \infty$. This is formally the same as equation (S26), with a complex consumption rate $\gamma(r) \to \gamma(r) + i\omega_n$, and the formal solution parallels that found in section III A 2 above:

$$\begin{aligned} \ln \phi_n(r) = \ln \phi_n(R) + \int_R^r \sqrt{\frac{i\omega_n + \gamma(r')}{D}} \frac{K_0'\left[\sqrt{i\omega_n + \gamma(r')/D}\, r'\right]}{K_0\left[\sqrt{i\omega_n + \gamma(r')/D}\, r'\right]} dr' \\ = \ln \phi_n(R) - \int_R^r \sqrt{\frac{i\omega_n + \gamma(r')}{D}} \frac{K_1\left[\sqrt{i\omega_n + \gamma(r')/D}\, r'\right]}{K_0\left[\sqrt{i\omega_n + \gamma(r')/D}\, r'\right]} dr'. \end{aligned} \tag{S41}$$

The calculation of this expression requires an evaluation of the Bessel functions of the second kind with complex argument prior to the integration step [2].

[1] Abramowitz, M. & Stegun, I. A. *Handbook of Mathematical Functions* (Dover New York, 1972).

[2] Mechel, F. Calculation of the Modified Bessel Functions of the Second Kind with Complex Argument. *Mathematics of Computation* **20**, 407–412 (1966).
